# *Zanthoxylum bungeanum* Waste-Derived High-Nitrogen Self-Doped Porous Carbons as Efficient Adsorbents for Methylene Blue

**DOI:** 10.3390/molecules29081809

**Published:** 2024-04-16

**Authors:** Yuhong Zhao, Qi Zhang, Zhuhua Gong, Wenlin Zhang, Yun Ren, Qiang Li, Hongjia Lu, Qinhong Liao, Zexiong Chen, Jianmin Tang

**Affiliations:** 1 Chongqing Key Laboratory for Germplasm Innovation of Special Aromatic Spice Plants, Institute of Special Plants, College of Smart Agriculture, Chongqing University of Arts and Sciences, Chongqing 402160, China; zyh0101hyz@163.com (Y.Z.); 18183376283@163.com (Q.Z.); 15208482161@163.com (Z.G.); reny1989@sina.com (Y.R.); liqiangxj@163.com (Q.L.); aaluhongjia@163.com (H.L.); lqhwisdom@126.com (Q.L.); chenzexiong1979@163.com (Z.C.); tangjmjy@163.com (J.T.); 2 College of Biology and Food Engineering, Chongqing Three Gorges University, Chongqing 404199, China

**Keywords:** *Zanthoxylum bungeanum* waste, N self-doped porous carbons, adsorbent, methylene blue

## Abstract

In this study, we prepared high-nitrogen self-doped porous carbons (NPC1 and NPC2) derived from the pruned branches and seeds of *Zanthoxylum bungeanum* using a simple one-step method. NPC1 and NPC2 exhibited elevated nitrogen contents of 3.56% and 4.22%, respectively, along with rich porous structures, high specific surface areas of 1492.9 and 1712.7 m^2^ g^−1^ and abundant surface groups. Notably, both NPC1 and NPC2 demonstrated remarkable adsorption abilities for the pollutant methylene blue (MB), with maximum monolayer adsorption capacities of 568.18 and 581.40 mg g^−1^, respectively. The adsorption kinetics followed the pseudo-second-order kinetics and the adsorption isotherms conformed to the Langmuir isotherm model. The adsorption mechanism primarily relied on the hierarchical pore structures of NPC1 and NPC2 and their diverse strong interactions with MB molecules. This study offers a new approach for the cost-effective design of nitrogen self-doped porous carbons, facilitating the efficient removal of MB from wastewater.

## 1. Introduction

Methylene blue (MB) is a common cationic soluble dye, widely utilized in the industries such as paper, textile, leather, and paint [1]. However, its widespread use has led to its substantial accumulation in aquatic environments, posing serious environmental and health risks due to its strong bioaccumulation potential, toxicity, resistance to degradation, carcinogenicity, and teratogenicity [2]. The direct discharge of MB into water bodies can disrupt ecological balance, harm aquatic life, endanger human health, and contribute to eutrophication [2,3]. Therefore, the effective removal of MB from industrial wastewater is essential before discharge. Currently, various methods, such as adsorption, photocatalysis, biodegradation, and chemical oxidation, are being employed for MB wastewater treatment [4], with adsorption being preferred owing to its cost-effectiveness, ease of regeneration, and high efficiency [5]. While a range of adsorbents, including carbon materials, hydrogels, polymers, metal nanomaterials, metal-organic frameworks and their derivatives, have been investigated for dye adsorption [6], most adsorbents encounter limitations such as low adsorption performance, potential secondary contamination, high cost, and complex manufacturing processes, which hinder their widespread application [7]. Therefore, there is a pressing need to develop efficient, eco-friendly, cost-effective, and readily available materials for MB removal.

Porous carbon has emerged as a promising adsorbent for MB wastewater treatment owing to its hierarchical porous structure [8]. The maximum monolayer adsorption capacities of MB onto porous carbons derived from eggshell membranes, coal slime, pineapple peel, pepper stalks and *Myristica fragrans* shell were 110.38, 125, 165.17, 178.4121 and 346.85 mg g^−1^, respectively [3,4,9,10,11]. To enhance the adsorption capacity of porous carbon, heteroatoms, particularly N atoms, have been introduced on the surface through doping [12]. N-doping alters the charge distribution of porous carbon and increases the number of defective carbon sites, thereby enhancing its adsorption capacity for pollutants [13,14]. Additionally, N-doping introduces N-containing functional groups, thereby improving the surface hydrophilicity of porous carbon [12]. Recent studies have demonstrated the effectiveness of N-doped carbon derived from sucrose and melamine, with a high specific surface area of 1417.4 m^2^ g^−1^ and a maximum MB adsorption capacity of 454.57 mg g^−1^ [15]. Jiang et al. [16] examined the adsorption of MB by N-doped porous carbon materials derived from macroalgae, attributing the excellent adsorption capacity to graphitic N sites with high electronegativity. Moreover, Li et al. [17] and Lv et al. [18] reported relatively high adsorption capacities of N-doped porous coral biochar and pine nut shell porous C (499.3 and 766.9 mg g^−1^, respectively) for MB.

Commonly, N atoms are incorporated into porous carbon through post-treatment N doping, involving the use of N-containing substances such as urea, ammonia, and melamine to treat the raw material or carbon material and introduce N [17]. However, this method has certain limitations, including high cost, low N-doping efficiency, and complexity [19,20]. In contrast, in situ N doping offers a simpler, more environmentally friendly, and efficient alternative. This method involves introducing N atoms into the porous carbon skeleton during the preparation of carbon materials by directly carbonizing the N-containing precursors [14,21]. Importantly, the use of N-containing precursor materials can ensure a more uniform distribution of N [22]. N is naturally present in biomass, enabling the production of N self-doped carbon materials [23]. Recently, various N self-doped porous carbons have been synthesized from sources such as pine wood [24], *Platanus acerifolia* (Aiton) fruit [23], palm flower [25], poplar catkin [21], garlic peel [26] and water hyacinth [27]. However, the N content of the porous carbons is often insufficient, limiting their practical application. Therefore, there is need to explore other inexpensive biomass sources to prepare high-N self-doped porous carbons.

**Figure 1:**
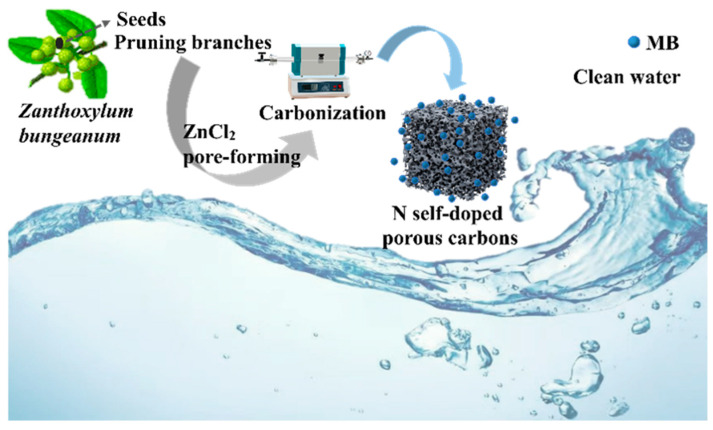
Schematic depiction of the synthesis and application of N self-doped porous carbons.

*Zanthoxylum bungeanum* (*Z. bungeanum*) is a small perennial deciduous tree belonging to the Rutaceae family and is valued for its use as a spice, food condiment, and medicinal herb. It holds considerable medicinal and culinary importance worldwide [28]. China, a major producer of *Z. bungeanum*, currently cultivates it over an area of approximately 73.5 thousand km^2^, and this cultivation has established it as a distinctive and thriving industry within the country [29]. During the planting and processing of *Z. bungeanum*, a large number of pruned branches (more than 2/3 of the crown weight) and seeds waste (about 1 million tons) are produced annually [30,31]. However, there has been no satisfactory utilization of *Z. bungeanum* waste till date. *Z. bungeanum* pruning branches and seeds are rich in carbon and N, making them excellent precursors for the preparation of high-N self-doped porous carbons [32]. To our knowledge, there have been few studies on N self-doped porous carbons derived from *Z. bungeanum* pruning branches and seeds as adsorbents for MB. In this study, N self-doped porous carbons were prepared using *Z. bungeanum* pruning branches and seeds as precursors with ZnCl_2_ as the pore-forming agent (Figure 1). And the preparation conditions, morphology, structure, chemical composition, and MB adsorption performance of the prepared porous carbon, as well as the underlying adsorption mechanism, were investigated.

## 2. Results and Discussion

### 2.1. Morphology and Structural Characterizations of N Self-Doped Porous Carbons

**Figure 2:**
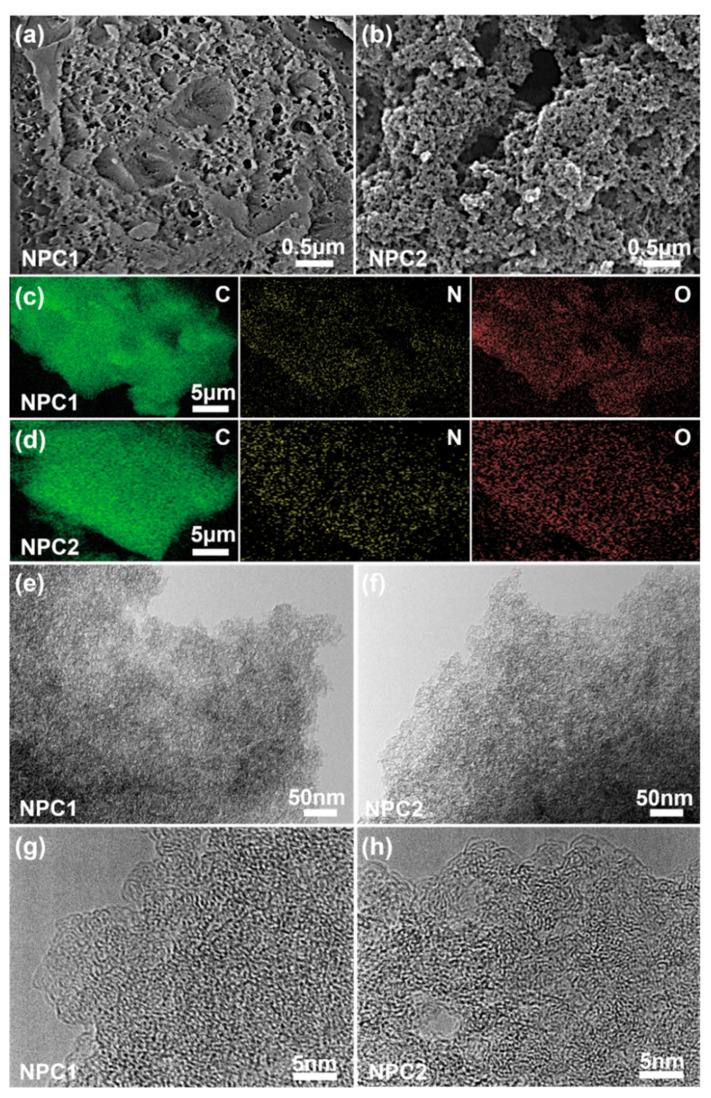
(**a**,**b**) SEM images, (**c**,**d**) EDS mappings and (**e**–**h**) TEM images of NPC1 and NPC2.

Scanning electron microscopy (SEM), transmission electron microscopy (TEM), and energy-dispersive X-ray spectroscopy (EDS) mapping were utilized to investigate the morphology, structure, and elemental compositions of NPC1 and NPC2. As shown in Figure 2a,b, the surfaces of both NPC1 and NPC2 displayed numerous macropore structures formed via the interweaving of the carbon layers [6], with NPC2 exhibiting a more abundant macropore structure. EDS patterns revealed the uniform distribution of C, N, and O atoms across the surfaces of NPC1 and NPC2 structural matrices (Figure 2c,d). Notably, the N content of NPC2 was significantly higher than that of NPC1 (Figure 2c,d). Additionally, TEM images revealed mesoporous and microporous structures in NPC1 and NPC2 (Figure 2e–h), alongside disordered turbine layer structures in their amorphous characteristics [14].

**Figure 3:**
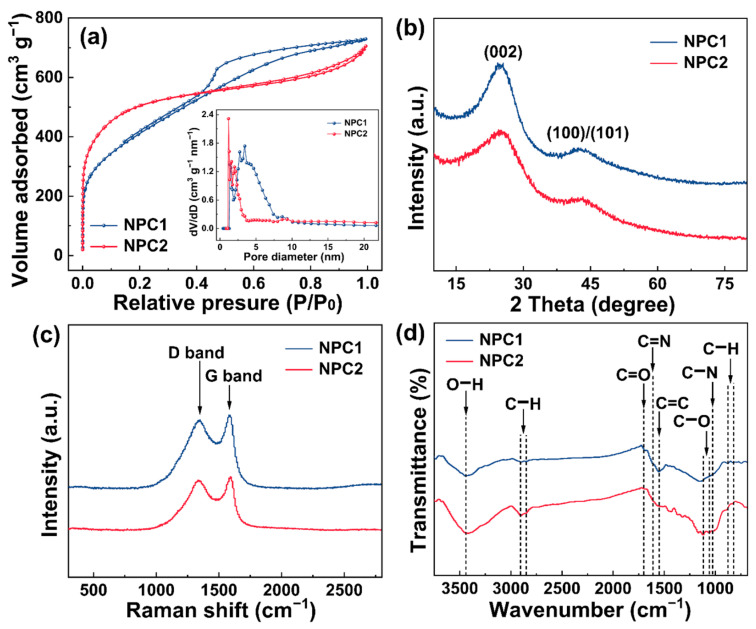
(**a**) N_2_ adsorption–desorption isotherms and DFT pore size distribution curves, (**b**) XRD patterns, (**c**) Raman spectra, and (**d**) FTIR spectra of NPC1 and NPC2.

To illustrate the textural properties of as-prepared NPC1 and NPC2, N_2_ adsorption–desorption isotherms were obtained. As depicted in Figure 3a, both NPC1 and NPC2 exhibited type I/IV isothermal characteristics. The isotherms of NPC1 and NPC2 rose sharply at low pressures P/P_0_ < 0.05, signifying the presence of micropores [13]. As pressure (P/P_0_ > 0.4) increased, a large hysteresis loop was observed in NPC1, indicating the presence of massive mesopores [27]. Conversely, NPC2 exhibited a small and narrow hysteresis loop, indicating fewer mesopores [33]. Moreover, an analysis of pore size distributions revealed the presence of abundant microporous and mesoporous structures in both NPC1 and NPC2 (inset of Figure 3a). Combined with the SEM and TEM results, it can be confirmed that the prepared porous carbons possessed rich hierarchical pore structures [6]. In addition, the BET-specific surface areas of NPC2 (1712.7 m^2^ g^−1^) were higher than those of NPC1 (1492.9 m^2^ g^−1^). However, the total pore volume of NPC1 (1.01 cm^3^ g^−1^) was slightly higher than that of NPC2 (0.85 cm^3^ g^−1^), exceeding those of the reported carbon materials (Table S1).

X-ray diffraction (XRD) analysis was conducted to examine crystalline structures of NPC1 and NPC2. As illustrated in Figure 3b, NPC1 displayed two wide diffraction peaks at 2θ = 24.8° and 43.8°, while NPC2 exhibited peaks at 2θ = 24.9° and 44.6°. These peaks corresponded to the crystalline planes (002) and (100)/(101) of the graphite, respectively, indicating that NPC1 and NPC2 existed as amorphous carbon with some degree of graphitization, which provided abundant adsorption sites for dyes [34,35]. Raman spectroscopy was employed to analyze the degrees of graphitization and disordered structures of NPC1 and NPC2. The disordered carbon (D band) and graphitized carbon (G band) at 1340 and 1584 cm^−1^ for NPC1 and at 1340 and 1592 cm^−1^ for NPC2, respectively (Figure 3c), were consistent with the XRD results, indicating amorphous carbon and graphitized carbon, respectively [9,33]. I_D_/I_G_ value of NPC2 (0.94) was higher than that of NPC1 (0.92), suggesting a relatively high graphitization degree for NPC2 [36].

The surface functional groups of NPC1 and NPC2 were identified using Fourier transform infrared (FTIR) spectroscopy. As shown in Figure 3d, NPC1 exhibited characteristic bands attributed to O–H (hydroxyl), C–H (methyl and methylene), C=O (ester, ether, and phenol), C=N (Schiff base), C=C (benzene ring), and C–H (stretching vibration of the benzene ring) at 3443 cm^−1^, 2900 and 2849 cm^−1^, 1684 cm^−1^, 1615 cm^−1^, 1551 cm^−1^, 878 and 822 cm^−1^, respectively [8,14,37,38]. NPC2 displayed absorption peaks at 3419 cm^−1^, 2904 and 2851 cm^−1^, 1699 cm^−1^, 1120 and 1057 cm^−1^, 1025 cm^−1^, representing O–H, C–H, C=O, C–O, and C–N groups, respectively [14,33,39]. These analyses revealed the presence of rich O–H, C–H, C=O, C=C, C–O, C=N, and C–N on the surfaces of NPC1 and NPC2, which facilitate MB adsorption through electrostatic attractions, hydrogen bonding, and π–π dispersion interactions [12,40].

**Figure 4:**
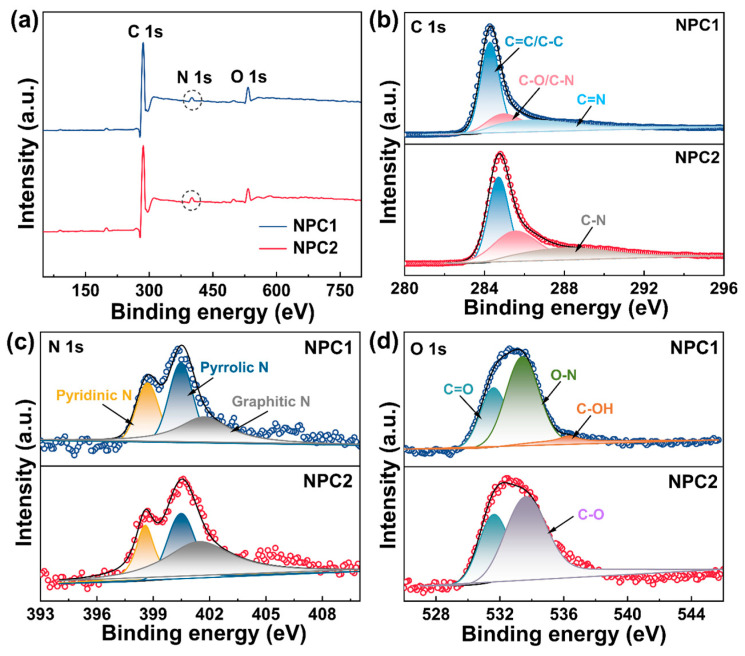
(**a**) XPS survey spectra of NPC1 and NPC2; (**b**) C 1s, (**c**) N 1s, and (**d**) O 1s high-resolution spectra of NPC1 and NPC2.

X-ray photoelectron spectroscopic (XPS) analysis was employed to further determine the chemical compositions of NPC1 and NPC2. As shown in Figure 4a, NPC1 and NPC2 contained C, N, and O. NPC2 exhibited a higher N content (4.22%) compared to NPC1 (3.56%) (Figure S2), exceeding that of other N self-doped porous carbons (Table S2). The N likely originated from alkaloids and nucleic acids in *Z. bungeanum* branches and proteins in seeds [26,32,41]. High-resolution C 1s spectra revealed peaks corresponding to C–C/C=C at 284.8 eV, C–O/C–N at 285.5 eV, and C=N at 287 eV for NPC1, and corresponding to C–C/C=C at 284.7 eV, C–O/C–N at 285.5 eV, and C–N at 288.1 eV for NPC2 (Figure 4b) [13,27]. Additionally, the high-resolution N 1s spectra displayed three peaks of NPC1 at 398.7, 400.48, and 401.7 eV or NPC2 at 398.57, 400.48, and 401.4 eV, corresponding to pyridinic N, pyrrolic N and graphitic N, respectively (Figure 4c) [42]. Particularly, pyridinic N provided electrons for π, thereby enhancing charge transfer as an efficient ion-absorbing substance [27], while graphitic N contributed to greater adsorption sites owing to its high thermal stability [6]. The O 1s spectra showed peaks corresponding to C=O at 531.6 eV, O–N at 533.4 eV, and C–OH at 536.3 eV for NPC1 [9,14], and corresponding to C=O at 531.6 eV and C–O at 533.6 eV for NPC2 (Figure 4d) [5,33]. These findings indicated that O-containing groups, such as –OH and –COOH, existed in NPC1 and NPC2. Importantly, these functional groups conferred hydrophilicity on carbon materials, and carboxyl groups had a high electron cloud density and could easily form hydrogen bonds, which was favorable for the adsorption of hydrophilic dyes [20].

### 2.2. Adsorption Performance of N Self-Doped Porous Carbons towards MB

In this study, NPC1 and NPC2 were prepared via ZnCl_2_ pore formation, with the mass ratio (MR) of ZnCl_2_ to raw material, activation temperature (T), and time (t) influencing their adsorption performance toward MB. Figure S1a illustrated that the equilibrium adsorption capacity (*q*_e_) of NPC1 and NPC2 toward MB at equilibrium increased with rising MR, reaching maximum values of 495.55 and 498.12 mg g^−1^ at a ratio of 3:1, respectively. This increase was likely due to the enhancement of the oxidative degradation and catalytic dehydration of the raw materials by ZnCl_2_, leading to the formation of abundant microporous and mesoporous structures and an increase in *q*_e_ [43]. A low MR favored the generation of micropores, while a high MR resulted in the formation of mesopores through a vigorous reaction between ZnCl_2_ and carbon [44]. However, the *q*_e_ values decreased to 476.87 and 464.19 mg g^−1^ for NPC1 and NPC2, respectively, as the MR increased to 4:1, possibly due to excessive ZnCl_2_ causing the micropores to collapse into mesopores and macropores [37]. With increasing activation temperature, the *q*_e_ values of NPC1 and NPC2 initially increased and then decreased (Figure S1b). The highest *q*_e_ for NPC1 and NPC2 was achieved at 500 °C and 600 °C, respectively, after which *q*_e_ gradually decreased. Elevated temperatures facilitated the formation of micropores and mesopores, thereby increasing the number of active sites and, thus, *q*_e_ increased [9]. However, excessively high temperatures could lead to the collapse or breakage of pore structures, resulting in a decrease in *q*_e_ [8]. Similarly, the *q*_e_ of NPC1 and NPC2 increased with increasing activation time up to 1 h, but decreased thereafter (Figure S1c). Initially, during carbonization, pore structures continued to form on the carbon surface, leading to an increase in *q*_e_ [44]. However, prolonged activation times could destroy pore structures and reduce the specific surface area of carbon, resulting in *q*_e_ decreasing [9].

**Figure 5:**
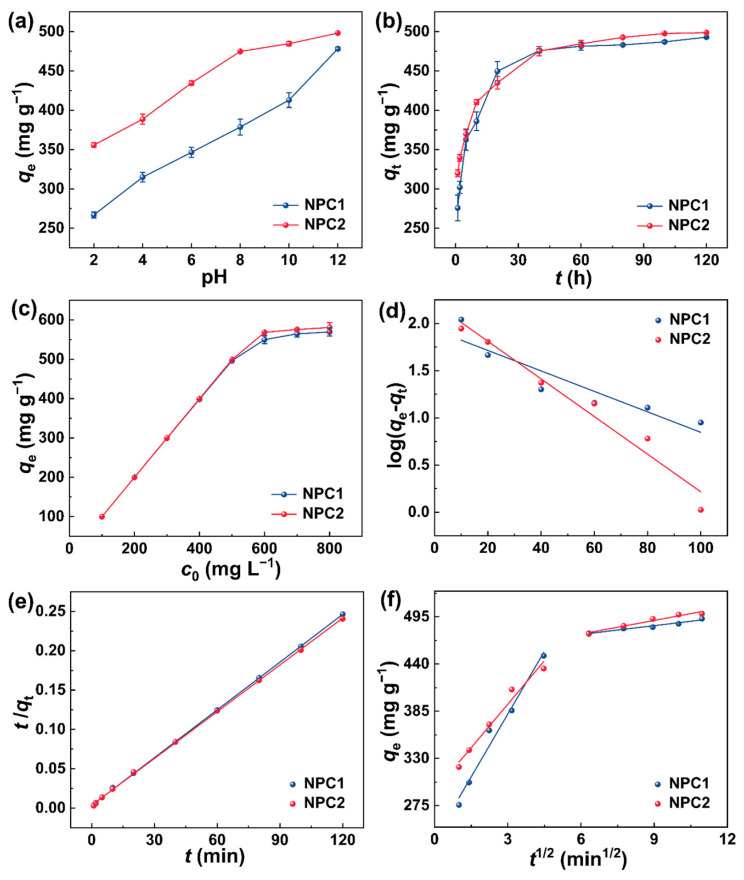
Changes in the *q*_e_ of NPC1 and NPC2 for MB with (**a**) pH (*c*_0_: 500 mg L^−1^, *T*: 25 °C, *t*: 1 h), (**b**) *t* (*c*_0_: 500 mg L^−1^, pH: 12, *T*: 25 °C), (**c**) *c*_0_ (pH: 12, *T*: 25 °C, *t*: 1 h) (**d**) PFO and (**e**) PSO kinetics models, (**f**) intraparticle diffusion model.

The solution pH is a crucial factor influencing the adsorption process, affecting the charge distribution of dye molecules and the adsorbent surface [42]. As the pH increased from 2 to 12, the *q*_e_ of NPC1 and NPC2 for MB also increased (Figure 5a). According to the previous report, cationic species were the predominant MB species in solutions [6]. And the surfaces of NPC1 and NPC2 were negatively charged at pH > pHzc_N_ (NPC1: 3.47; NPC2: 4.53, Figure S3a,b), respectively. When the pH rose from 3.47 or 4.53 to 12, the functional groups on the carbons’ surface were deprotonated, electrostatic attraction with MB molecules enhanced gradually and resulted in an increase in *q*_e_ [4,45]. As shown in Figure 5b, NPC1 and NPC2 rapidly adsorbed MB within 1 min, with the adsorption capacities reaching 275.79 and 319.87 mg^−1^, respectively. This rapid adsorption was attributed to the presence of mesopores and macropores in the porous carbon structure, facilitating the capture of MB molecules [15]. However, as adsorption progressed, the rate gradually declined because the adsorption sites were occupied [10], reaching dynamic equilibrium after 60 min. Additionally, the *q*_e_ values increased with increasing initial concentration of MB solution (*c*_0_) (Figure 5c), as higher concentrations created greater driving forces from the concentration gradient [46]. The adsorption temperature also influenced the adsorption reactions and equilibrium capacity [47]. Figure S4 demonstrates that as temperature increased from 20 to 65 °C, the *q*_e_ values for NPC1 and NPC2 continuously increased. This indicated that the adsorption processes were endothermic, with the elevated temperature enhancing the mobility of the MB solution, allowing MB molecules to interact more effectively with active sites on the carbons’ surfaces, thereby improving *q*_e_ [6,10].

The process of adsorption of MB by NPC1 and NPC2 was assessed using pseudo-first-order (PFO) and pseudo-second-order (PSO) kinetics, as well as intra-particle diffusion models, expressed by the following equations:

(1) $$\log\left(q_{e}-q_{t}\right)=\log q_{e}-\frac{k_{1}t}{2.303}$$

(2) $$\frac{t}{q_{t}}=\frac{1}{k_{2}q_{e}^{2}}+\frac{t}{q_{e}}$$

(3) $$q_{t}={\;k}_{i}t^{1/2}+c_{i}$$

Here, *q*_e_ (mg g^−1^) is the equilibrium adsorption capacity, *q*_t_ (mg g^−1^) is the adsorption capacity at a given time, *t* (min) is the adsorption time, *k*_1_ (min^−1^) and *k*_2_ (min^−1^) are the rate constants for PFO and PSO kinetics, respectively, *k*_i_ (mg g^−1^ h^−1/2^) represents the diffusion rate constant in the particle, and *c*_i_ is a constant (mg g^−1^).

The linear fittings of the kinetic data sets are depicted in Figure 5d,e, with the corresponding kinetics parameters summarized in Table 1. Notably, for both NPC1 and NPC2, the PSO model coefficients (*R*^2^ > 0.999) exceeded those of the PFO model, and lower root mean square error (RMSE) was lower than that of the PFO model. Furthermore, the difference in experimental data (*q*_e_, _exp_) and the calculated equilibrium adsorption capacities (*q*_e_, _cal_) (∆*q*) of PFO were much higher than those of PSO, indicating that the adsorption process adheres to the PSO kinetics model. Furthermore, the chi-square (χ^2^) of PSO was lower than that of PFO, demonstrating that the adaptability of PSO was better [48,49]. Therefore, the primary mechanism driving the adsorption of MB by NPC1 and NPC2 likely involved chemisorption, characterized by electron transfer or sharing between the adsorbent and the adsorbate [6,50].

The intra-particle diffusion model was employed to explore the potential rate-determining step of the adsorption process. The linear fittings were illustrated in Figure 5f, with the corresponding parameters summarized in Table 2. The adsorption of MB by NPC1 and NPC2 followed two distinct steps. Initially, membrane diffusion facilitated the migration of MB molecules from the fluid phase to the outer surface of the porous carbons, constituting a surface mass transfer process [47]. Subsequently, intra-particle diffusion occurred, involving the diffusion of MB molecules from the exterior to the interior of the porous carbons [51]. Notably, *k*_i1_ significantly surpassed *k*_i2_, which indicated that intra-particle diffusion proceeded at a slower pace. Furthermore, *c*_i1_ did not intersect the origin, suggesting the involvement of other mechanisms, such as liquid film diffusion in the adsorption process [13].

The adsorption characteristics of NPC1 and NPC2 toward MB were further examined using the Langmuir, Freundlich, and Temkin isotherm models. The main features of the Langmuir isotherm were evaluated using the dimensionless separation factor (Q), with the linear equations expressed as follows:

(4) $$\frac{c_{e}}{q_{e}}=\frac{c_{e}}{q_{m}}+\frac{1}{bq_{m}}$$

(5) $$\log q_{e}=\log k+\frac{1}{n}\log c_{e}$$

(6) $$q_{e}=B\ln K_{T}+B\ln c_{e}$$

(7) $$Q=\frac{1}{1\;+\;bc_{0}}$$

Here, *q*_m_ (mg g^−1^) is the maximum monolayer adsorption capacity; *c*_e_ (mg L^−1^) is the equilibrium concentration of MB solution; *b* (L mg^−1^) is the Langmuir adsorption constant; *k* (mg g^−1^(L mg^−1^)^1/n^) and n are the constants related to the temperature and system, respectively; *B* (J mol^−1^) represents the Temkin isotherm constants; *K*_T_ (L g^−1^) is related to the highest binding energy; and Q is the isotherm type: non-reversible (Q = 0), favorable (0 < Q < 1), linear (Q = 1) or non-favorable (Q > 1).

**Figure 6:**
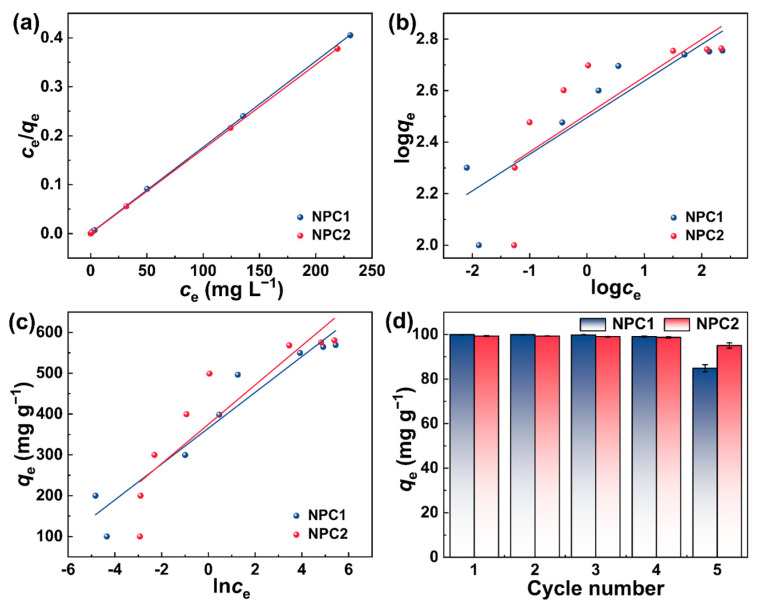
(**a**) Langmuir, (**b**) Freundlich, and (**c**)Temkin isotherm models, (**d**) the reusability of NPC1 and NPC2 for MB adsorption (*c*_0_: 100 mg L^−1^, pH: 12, *T*: 25 °C, 1 h).

The linear fittings of the adsorption isotherm models and the corresponding parameters of NPC1 and NPC2 were presented in Figure 6a–c and summarized in Table 3. All the *R*^2^ values (>0.999) of the Langmuir model surpassed those of the Freundlich and Temkin models. Moreover, the RMSE and χ^2^ of the Langmuir model displayed lower values compared to Freundlich and Temkin, indicating that NPC1 and NPC2 adsorption processes were more consistent with the Langmuir model. Similar results were obtained in other studies [11,48,52]. This revealed that monolayer adsorption occurred on the porous carbons’ surface with a uniform distribution of adsorption sites and adsorption energy, without interactions between the adsorbed MB molecules [42]. In addition, from the Langmuir model, the maximum monolayer adsorption capacity (*q*_m_) of NPC2 was 581.40 mg g^−1^, exceeding that of NPC1(568.18 mg g^−1^), which was higher than that of other waste-based porous carbons (Table S3). Moreover, the calculated Q values for MB were 0.0011 and 0.0006, ranging from 0–1, indicating the favorable adsorption of MB by NPC1 and NPC2 [18].

The cyclic stability of N self-doped porous carbon is crucial for reducing production costs. As shown in Figure 6d, the *q*_e_ values of NPC1 and NPC2 for MB decreased slightly after five cycles, potentially due to channel clogging [53]. Nevertheless, the MB removal rates of NPC1 and NPC2 remained above 84.8% and 95%, respectively, indicating their outstanding reusability. According to the previous reports [6,52], the good regeneration of NPC1 and NPC2 confirmed their highly stable structures.

### 2.3. Underlying Adsorption Mechanism of MB by N Self-Doped Porous Carbons

**Figure 7:**
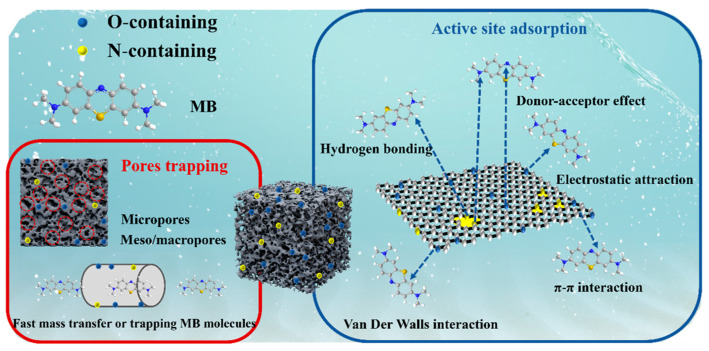
Diagram illustrating the mechanism diagram of the adsorption of MB by the prepared N self-doped porous carbons.

A plausible mechanism explaining the efficient adsorption of MB by the prepared N self-doped porous carbon was proposed (Figure 7). The above analysis indicated that the prepared N self-doped porous carbons possess rich pore structures and abundant functional groups (Figure 2, Figure 3d and Figure 4). First, the hierarchical porous structure of the carbons provided ample active adsorption sites for MB molecules. Notably, macropores and some mesopores with larger sizes offered favorable pore channels for MB molecules, facilitating the mass transfer process of MB [4]. Most mesopores and micropores can trap more MB molecules, thereby enhancing the adsorption capacity of the porous carbons [9]. Simultaneously, the powerful, strong electrostatic attraction between the negatively charged functional groups (–COOH and –OH) on the carbon surface and the positively charged MB molecules drive the adsorption process [40,54]. Importantly, the N-containing groups in the porous carbons can provide for the electron-deficient sites of MB molecules via the “donor–acceptor effect” [51]. The presence of N atoms in the carbons adjusted the electronic distribution of C atoms, thereby enhancing the π–π dispersion interaction between the porous carbons and the benzene ring of MB molecules [17,55]. Additionally, hydrogen bonding between the H atoms on the porous carbon surface and the N atoms on the MB molecules may be an important and crucial factor in the adsorption [6,56]. Moreover, van der Waals forces contributed to the adsorption process [41]. Consequently, the adsorption mechanism of the N self-doped porous carbons was considered to involve the abundant porous structures within the porous carbons and their various strong interactions with the MB dye.

## 3. Materials and Methods

### 3.1. Materials and Reagents

The *Z. bungeanum* pruning branches and seeds utilized in this experiment were sourced from a *Z. bungeanum* base located in Yongchuan, Chongqing, China. MB was procured from Shanghai Aladdin Biochemical Technology Co., Ltd. (Shanghai, China). Analytical-grade ZnCl_2_, ethanol, and other reagents were acquired from Chengdu Kelong Chemical Reagent Co. (Chengdu, China).

### 3.2. Preparation of N Self-Doped Porous Carbons

Initially, the *Z. bungeanum* pruning branches and seeds were dried, ground into a powder with a particle size of 60 mesh, and stored in a dryer for subsequent use. To begin the preparation process, a certain amount of ZnCl_2_ and 1 g of pruning branches or seed powders were dispersed in 50 mL ultrapure water at various ratios (0.5, 1, 2, 3, 4) with vigorous stirring for 60 min at 25 °C. Subsequently, the mixtures were dried in a hot air oven at 60 °C. The dried mixtures were then heated to different activation temperatures (300, 400, 500, 600, and 700 °C) at a heating rate of 5 °C min^−1^ in a tube furnace under high purity N_2_ atmosphere. They were maintained at different activation times (0.5, 1, 1.5, 2, and 2.5 h) to prepare the crude porous carbon products. Finally, the crude products were ground, crushed, and washed with 1 mol L^−1^ HCl and pure water, combined with an ultrasound to remove impurities. They were then dried at 60 °C to obtain the purified N self-doped porous carbon derived from pruning branches (NPC1) and seeds (NPC2). Based on the adsorption performance of MB, the optimal preparation conditions for NPC1 were determined to be as follows: MR = 3:1, T = 500 °C, t = 1 h. And those for NPC2 were as follows: MR = 3:1, T = 600 °C, t = 1 h.

### 3.3. Characterization of N Self-Doped Porous Carbons

The morphologies of NPC1 and NPC2 were examined using a Hitachi SU8220 field-emission scanning electron microscope (Hitachi, Hitachi, Japan) operated at 20 kV and a Hitachi JEOL-2100 transmission electron microscope (Hitachi, Japan) operated at 200 kV. XRD patterns were obtained using an Ultima IV X-ray diffractometer (Rigaku, Tokyo, Japan) with Cu Kα radiation (λ = 0.1542 nm). FTIR spectra were recorded using a Nicolet 6700 spectrophotometer (Thermo Fisher, Waltham, MA, USA). N_2_ adsorption–desorption isotherms were measured using a Quadrasorb instrument (Quantachrome, Boynton Beach, FL, USA), and the samples were degassed for 12 h at 120 °C in an internal oven of the apparatus. Data were analyzed using the ASAP 2460 Quantachrome software. Raman spectra were recorded on a DXR Raman spectroscopy system (Thermo Fisher Scientific). XPS data were obtained using an Escalab 250Xi X-ray photoelectron spectrometer (Semmerfeld Technologies, Jacksonville, FL, USA). The pHzc_N_ value at ΔpH = 0 was determined from the intersection point of the initial pH and final pH curves.

### 3.4. Adsorption of MB by N Self-Doped Porous Carbons

Adsorption experiments were conducted by adding 10 mg of NPC1 or NPC2 to 10 mL of the MB dye solution. The mixtures were then shaken on a shaker at 200 rpm under various conditions, including the solution pH (2–12, adjusted by 0.04 mol L^−1^ H_3_PO_4_, H_3_BO_3_ and CH_3_COOH mixed solution and 0.2 mol L^−1^ NaOH solution), adsorption time (t: 0–120 min), adsorption temperature (T: 20–65 °C), and the initial concentration of MB (*c*_0_: 100–800 mg L^−1^). After adsorption, the solution was centrifuged at 5000 rpm, and the absorbance of the supernatant was measured at 664 nm using a UV–Vis spectrophotometer (Beijing Persee General Instrument Co., Beijing, China). The final MB concentration was calculated using an MB standard curve of MB. The adsorption capacities were calculated using the following formula:

(8) $$q_{e}=(c_{0}-c_{e})\;\times \;\frac{V}{m}$$

Here, *c*_0_ (mg L^−1^) is the initial concentration of MB solution, *V* (L) is the volume of solution, and *m* (g) is the mass of N self-doped porous carbons.

Three replicates of each sample were performed to ensure the accuracy of the experimental data. The χ^2^, ∆*q* and RMSE were utilized to evaluate the accuracy of the used linear kinetics and isotherm models [57].

### 3.5. Recyclability of N Self-Doped Porous Carbons

In separate experiments, 10 mg of NPC1 or NPC2 were introduced into 10 mL of a 100 mg L^−1^ MB solution (pH = 12), and the mixtures were shaken at 200 rpm and 25 °C for 60 min. After adsorption, the mixtures were centrifuged, and the absorbance of the supernatant was measured. Subsequently, 10 mL ethanol (pH = 4) was added to the NPC1 and NPC2 samples saturated 10 min. The mixtures were then centrifuged at 5000 rpm to remove the supernatant. This process was repeated five times.

## 4. Conclusions

In this study, the N self-doped porous carbons of NPC1 and NPC2 were successfully synthesized using *Z. bungeanum* pruning branches and seeds, respectively. The prepared NPC1 and NPC2 exhibited high N contents, well-developed pore structures, abundant surface groups, and large specific surface areas. They demonstrated excellent adsorption ability for MB dye with remarkable recyclability. The adsorption process fitted the PSO kinetics and Langmuir models. The adsorption mechanism was attributed to the combined effect of hierarchical pores in NPC1 and NPC2 and their diverse strong interactions with MB molecules. This research offers a novel approach for the valuable utilization of *Z. bungeanum* waste and the development of high-quality adsorbents for wastewater treatment.

## Figures and Tables

**Table 1:** Parameters of the kinetics models (PFO and PSO).

Materials		NPC1	NPC2
	*q*_e, exp_ (mg g^−1^)	492.9	498.7
PFO	*q*_e, cal_ (mg g^−1^)	85.4	170.22
	*k*_1_ (min^−1^)	0.02	0.05
	*R* ^2^	0.8264	0.9770
	∆*q*	407.5	328.48
	RMSE	0.1387	0.1010
	χ^2^	0.0289	0.0131
PSO	*q*_e, cal_ (mg g^−1^)	495.0	505.05
	*k*_2_ (g mg^−1^ min^−1^)	0.0012	0.0011
	*R* ^2^	0.9998	0.9996
	∆*q*	−2.1	−6.35
	RMSE	0.0001	0.0015
	χ^2^	1.2403 × 10^−6^	2.7634 × 10^−6^

**Table 2:** Parameters of the intra-particle diffusion model.

Materials		NPC1	NPC2
First stage	*k*_i1_ (mg g^−1^ h^−1/2^)	48.80	33.76
	*c*_i1_ (mg g^−1^)	235.30	291.92
	*R* ^2^	0.9695	0.9649
	RMSE	9.3446	6.9376
	χ^2^	145.5373	80.2181
Second stage	*k*_i2_ (mg g^−1^ h^−1/2^)	3.41	5.30
	*c*_i2_ (mg g^−1^)	454.01	443.09
	*R* ^2^	0.9437	0.9476
	RMSE	1.1711	1.7522
	χ^2^	2.2858	5.1168

**Table 3:** Parameters of the Langmuir, Freundlich, and Temkin isotherm models.

Materials		NPC1	NPC2
Langmuir	*q*_m_ (mg g^−1^)	568.18	581.40
	*b* (L mg^−1^)	1.79	3.49
	*R* ^2^	0.9999	0.9999
	RMSE	0.0010	0.0006
	χ^2^	1.4298 × 10^−6^	4.6118 × 10^−7^
Freundlich	*k* (mg g^−1^(L mg^−1^)^1/n^)	312.73	321.99
	1/*n*	0.14	0.15
	*R* ^2^	0.7878	0.5847
	RMSE	0.1079	0.1530
	χ^2^	0.0155	0.0312
Temkin	*K*_T_ (L g^−1^)	316.83	264.42
	*B* (J mol^−1^)	43.92	48.18
	*R* ^2^	0.9190	0.7836
	RMSE	44.4434	74.8569
	χ^2^	2.6363 × 10^3^	7.4714 × 10^3^

## Data Availability

Data are contained within the article and Supplementary Materials.

## Supplementary Materials

The following supporting information can be downloaded at: https://www.mdpi.com/article/10.3390/molecules29081809/s1. Figure S1: Effect of the preparation parameters of (a) MR (T: 500 °C, t: 1 h), (b) T (MR: 3:1, t: 1 h), (c) t (MR: 3:1, T: 500 °C (NPC1), 600 °C (NPC2)) of NPC1 and NPC2 towards MB adsorption (*c*_0_: 500 mg L^−1^, pH=12, 1 h, 25 °C). Figure S2: The relative elemental contents of NPC1 and NPC2 from XPS. Figure S3: Zero charge point of NPC1 (a) and NPC2 (b). Figure S4: Changes in the *q*_e_ of NPC1 and NPC2 for MB with *T* (*c*_0_: 600 mg L^−1^, pH: 12, 1 h). Table S1: Comparison of the specific surface area and total pore volume of NPC1, NPC2 and other carbon materials. Table S2: Comparison of the surface N content of the obtained NPC1, NPC2 and other N self-doped carbon materials. Table S3: Comparison of the *q*_m_ of NPC1, NPC2 and other waste-based carbon materials. The references [58,59,60,61,62,63,64,65,66,67] are from the Supplementary Materials.
