# Supplementary material for: Zanthoxylum bungeanum Waste-Derived High-Nitrogen Self-Doped Porous Carbons as Efficient Adsorbents for Methylene Blue
Source: Molecules. 2024 Apr 16;29(8):1809. doi: 10.3390/molecules29081809 (PMC11052492; doi:10.3390/molecules29081809)
Supplement: Supplementary file 1 [file molecules-29-01809-s001.zip › molecules-2962795-supplementary.pdf]

Supplementary Materials

Zanthoxylum bungeanum Waste-Derived High-Nitrogen Self-Doped Porous Carbons as Efficient Adsorbents for Methylene Blue

Yuhong Zhao [1,2], Qi Zhang [1], Zhuhua Gong [1], Wenlin Zhang [1,*], Yun Ren [1], Qiang Li [1], Hongjia Lu [1], Qinhong Liao [1], Zexiong Chen [1] and Jianmin Tang [1]

[1] Chongqing Key Laboratory for Germplasm Innovation of Special Aromatic Spice Plants, Institute of Special Plants, College of Smart Agriculture, Chongqing University of Arts and Sciences, Chongqing 402160, China; zyh0101hyz@163.com (Y.Z.); 18183376283@163.com (Q.Z.); 15208482161@163.com (Z.G.); reny1989@sina.com (Y.R.); liqiangxj@163.com (Q.L.); aaluhongjia@163.com (H.L.); lqhwisdom@126.com (Q.H.L.); chenzexiong1979@163.com (Z.C.); tangjmjy@163.com (J.T.)

[2] College of Biology and Food Engineering, Chongqing Three Gorges University, Chongqing 404199, China

* Correspondence: zhangwenlin@cqwu.edu.cn

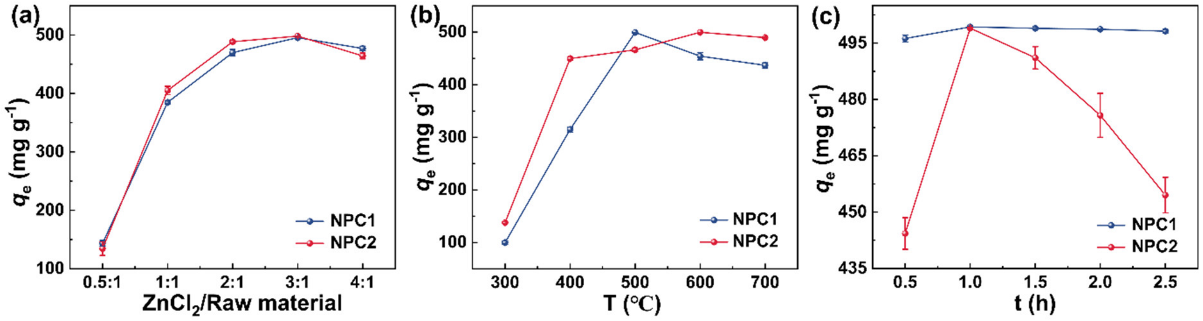

Figure S1. Effect of the preparation parameters of (a) MR (T: 500 °C, t: 1 h), (b) T (MR: 3:1, t: 1 h), (c) t (MR: 3:1, T: 500 °C (NPC1), 600 °C (NPC2)) of NPC1 and NPC2 towards MB adsorption (c_0: 500 mg L^{-1}, pH=12, 1 h, 25 °C).

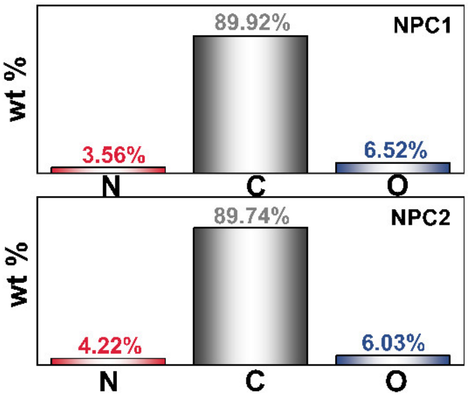

Figure S2. The relative elemental contents of NPC1 and NPC2 from XPS.

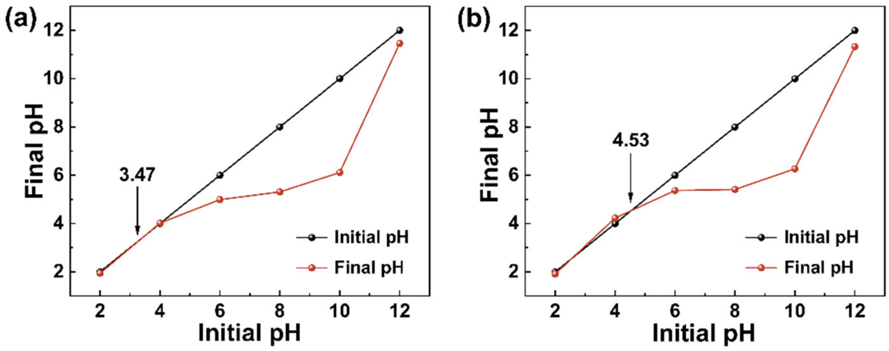

Figure S3. Zero charge point of of NPC1(a) and NPC2 (b).

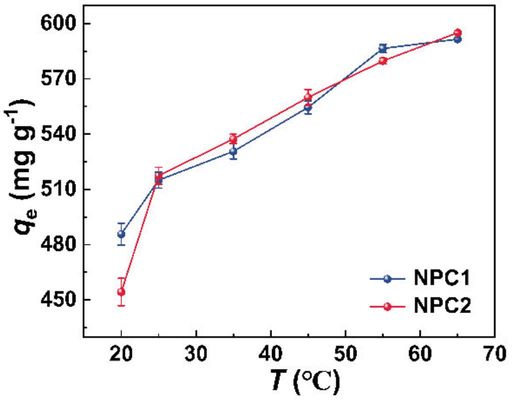

Figure S4. Changes in q_e of NPC1 and NPC2 for MB with T (c_0: 600 mg L^{-1}, pH: 12, 1 h).

Table S1. Comparison of specific surface area and total pore volume of NPC1, NPC2 and other carbon materials.

Carbon materials	S_{BET} (m^2 g^{-1})	V_T (m^3 g^{-1})	References
Groundnut shell activated carbon	412	0.322	[1]
Green solid waste N-self-doped porous carbon	632.76	0.35	[2]
Coconut shell magnetic activated carbon	747.71	0.42	[3]
Pomelo peel waste-derived porous carbon	939.4	0.62	[4]
Pineapple peel activated carbon	1160.07	0.544	[5]
Seaweed activated carbon	1238.491	/	[6]
Industrial alkali lignin N-doped porous carbon	1309	0.733	[7]
Myristica fragrans shell activated carbon	1462	0.873	[8]
**NPC1**	**1492.9**	**1.01**	**This work**
**NPC2**	**1712.7**	**0.85**	**This work**

Table S2. Comparison of surface N content of obtained of NPC1, NPC2 and other N-self-doped carbon materials.

N-self-doped carbon materials	N (at. %)	References
Lignin N, O-codoped porous carbon	1.07	[9]
N/S self-doping hierarchical porous carbon	1.24	[10]
Platanus acerifolia (Aiton) Willd. fruit-derived nitrogen-doped porous carbon	1.31	[11]
Garlic peels nitrogen self-doped porous carbon	1.77	[12]
Water hyacinth N-self-doped porous carbon	2.73	[13]
Honeycomb-like N/O self-doped hierarchical porous carbons derived from low-rank coal	2.9	[14]

N-self-doped porous carbon derived from animal-heart	3.03	[15]
Poplar catkin N-doped hierarchical porous carbon	3.16	[16]
Semi-coking wastewater derived N–O–S self-doped porous carbon	3.39	[17]
**NPC1**	**3.56**	**This work**
**NPC2**	**4.22**	**This work**

Table S3. Comparison of q_m of NPC1, NPC2 and other waste-based carbon materials.

Carbon materials	q_m (mg g^{-1})	References
Eggshell membrane porous biochar adsorbent	110.38	[18]
Coconut shell magnetic activated carbon	156.25	[3]
Pineapple peel activated carbon	165.17	[5]
Seaweed activated carbon	243.839	[6]
Myristica fragrans shell activated carbon	346.85	[8]
Iron doped activated carbon derived from *Delonix regia* barks	357.142	[19]
Heavy bio-oil porous biochar	411	[20]
Cyanobacteria-plastic composite carbon	490	[21]
**NPC1**	**568.18**	**This work**
**NPC2**	**581.40**	**This work**

References

1. Mani, D.; Elango, D.; Priyadharsan, A.; Al-Humaid, L. A.; Al-Dahmash, N. D.; Ragupathy, S.; Jayanthi, P.; Ahn, Y. H. Groundnut shell chemically treated with KOH to prepare inexpensive activated carbon: Methylene blue adsorption and equilibrium isotherm studies. *Environ. Res.* **2023**, *231(1)*, 116026.
2. Hao, R. N.; Ma, R. N.; Cao, Y. L.; Zhou, W. Y.; Chai, H. Facile fabrication of N-self-doped porous carbons from green solid waste for supercapacitors with high cycling stability and flexibility. *Mater. Today Commun.* **2022**, *33*, 104911.
3. Yağmur, H. K.; Kaya, İ. Synthesis and characterization of magnetic $ZnCl_2$-activated carbon produced from coconut shell for the adsorption of methylene blue. *J. mol. Struct.* **2021**, *1232*, 130071.
4. Zhang, W. L.; Liu, M. W.; Zhao, Y. H.; Liao, Q. H., Facile preparation of porous carbon derived from pomelo peel for efficient adsorption of methylene blue. *Molecules* **2022**, *27(10)*, 3096.
5. Rosli, N. A.; Ahmad, M. A.; Noh, T. U. Unleashing the potential of pineapple peel-based activated carbon: Response surface methodology optimization and regeneration for methylene blue and methyl red dyes adsorption. *Inorg. Chem. Commun.* **2023**, *155*, 111041.
6. Jiang, D.; Li, H. P.; Cheng, X. X.; Ling, Q. F.; Chen, H.; Barati, B.; Yao, Q. F. Abomohra, A.; Hu, X.; Bartocci, P.; Wang, S. A mechanism study of methylene blue adsorption on seaweed biomass derived carbon: From macroscopic to microscopic scale. *Process Saf. Environ.* **2023**, *172*, 1132-1143.
7. Zheng, L.; Wang, H. M.; Wang, X. H.; Kong, F. G.; Liu, Y.; Zhang, F. S. Fabrication of N-doped micro-mesoporous carbons from industrial alkali lignin with urea assistance for high-efficiency adsorption of methylene blue. *Ind. Crop. Prod.* **2023**, *203*, 117146.

8. Mariana, M.; Mistar, E. M.; Alfatah, T.; Supardan, M. D. High-porous activated carbon derived from Myristica fragrans shell using one-step KOH activation for methylene blue adsorption. *Bioresour. Technol. Rep.* **2021**, *16*, 100845.
9. Gong, L. Z.; Bao, A. G. L. High-value utilization of lignin to prepare N, O-codoped porous carbon as a high-performance adsorbent for carbon dioxide capture. *J. CO_2 Util.* **2023**, *68*, 102374.
10. Dong, K. M.; Liu, S.; Guo, F. Q.; Wang, J. J.; Tang, B.; Zhao, N. J.; Kong, L. W.; Zhang, Y. D. Facile synthesis of hierarchical porous carbon electrodes with 3D self-supporting structure and N/S self-doping for advanced energy storage device. *J. Energy Storage* **2023**, *72(A)*, 108218.
11. Li, Y.; Li, H. X.; Zhou, T. T.; Lai, Q.; Egabaierdi, G.; Chen, S. W.; Song, H. O.; Zhang, S. P.; Shi, C. F.; Yang, S. G.; He, H.; Zhang, X. Q. Platanus acerifolia (Aiton) Willd. fruit-derived nitrogen-doped porous carbon as an electrode material for the capacitive deionization of brackish water. *J. Environ. Chem. Eng.* **2023**, *11(3)*, 109914.
12. Liu, S.; Dong, K. M.; Guo, F. Q.; Qiao, Q. X.; Xu, L. Y.; Wang, J. J.; Kong, L. W.; Zhang, Y. D.; Chang, J. F.; Yan, W. J. Green synthesis of nitrogen self-doped porous carbons from waste garlic peels for high-performance supercapacitor applications. *J. Anal. Appl. Pyrol.* **2023**, *173*, 106063.
13. Zhang, X. k.; Ma, X. Q.; Yu, Z. S.; Yi, Y. J.; Lu, C. X.; Lu, X. L. Microwave-assisted two-step pyrolysis of water hyacinth for the preparation of N-self-doped porous carbon. *J. Anal. Appl. Pyrol.* **2023**, *173*, 106061.
14. Li, L.; Wei, X. Y.; Shao, C. W.; Yin, F.; Sun, B. K.; Liu, F. J.; Li, J. H.; Liu, Z. Q.; Zong, Z. M. Honeycomb-like N/O self-doped hierarchical porous carbons derived from low-rank coal and its derivatives for high-performance supercapacitor. *Fuel* **2023**, *331(1)*, 125658.
15. Yang, B. Y.; Gao, J. P.; Xie, M. H.; Zuo, S. S.; Kang, H.; Sun, Y.; Xu, X. Y.; Wang, W.; Gao, C. J.; Liu, Y.; Yan, J. N-self-doped porous carbon derived from animal-heart as an electrocatalyst for efficient reduction of oxygen. *J. Colloid Interf. Sci.* **2020**, *579(1)*, 832-841.
16. Chang, B. B.; Shi, W. W.; Yin, H.; Zhang, S. R. Poplar catkin-derived self-templated synthesis of N-doped hierarchical porous carbon microtubes for effective CO_2 capture. *Chem. Eng. J.* **2019**, *358*, 1507-1518.
17. Wang, X. J.; Wang, Y. F.; Yan, L.; Wang, Q.; Li, J.; Zhong, X.; Liu, Q. Q.; Li, Q. C.; Cui, S.; Xie, G. From pollutant to high-performance supercapacitor: Semi-coking wastewater derived N–O–S self-doped porous carbon. *Colloi. Surface. A* **2023**, *657(B)*, 130596.
18. Wu, T. T.; Yang, G. P.; Cao, J. X.; Xu, Z. W.; Jiang, X. H. Activation and adsorption mechanisms of methylene blue removal by porous biochar adsorbent derived from eggshell membrane. *Chem. Eng. Res. Des.* **2022**, *188*, 330-341.
19. Joshiba, G. J.; Kumar, P. S.; Rangasamy, G.; Ngueagni, P. T.; Pooja, G.; Balji, G. B.; Alagumalai, K.; El-Serehy, H. A. Iron doped activated carbon for effective removal of tartrazine and methylene blue dye from the aquatic systems: Kinetics, isotherms, thermodynamics and desorption studies. *Environ. Res.* **2022**, *215(3)*, 114317.
20. Zhang, H. Y.; Peng, B.; Liu, Q. Y.; Wu, C. S.; Li, Z. W. Preparation of porous biochar from heavy bio-oil for adsorption of methylene blue in wastewater. *Fuel Process. Technol.* **2022**, *238*, 107485.
21. Li, L. X.; Wang, J.; Jia, C.; Lv, Y.; Liu, Y. Co-pyrolysis of cyanobacteria and plastics to synthesize porous carbon and its application in methylene blue adsorption. *J. Water Process Eng.* **2020**, *39*, 101753.
